# An Alternative Approach to Plastic Recycling: Fabrication and Characterization of rPET/CA Nanofiber Carriers to Enhance Porcine Pancreatic Lipase Stability Properties

**DOI:** 10.1021/acsomega.3c07227

**Published:** 2024-07-09

**Authors:** Ceyhun IŞIK

**Affiliations:** Faculty of Science, Chemistry Department, Muğla Sıtkı Koçman University, Muğla 48000, Türkiye

## Abstract

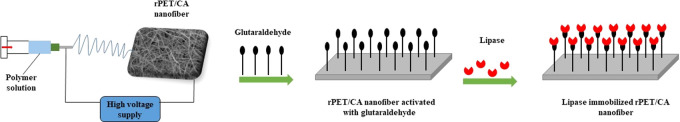

In response to the increasing demand for sustainable technologies, this study presents a novel approach to plastic recycling. In this study, a method was presented to produce nanofiber carriers by electrospinning using recycled poly(ethylene terephthalate) (rPET) obtained from wastewater bottles and cellulose acetate (CA). These carriers serve as a platform for immobilized porcine pancreatic lipase (PPL), aiming to enhance its stability. The production parameters for the rPET/CA nanofibers were found to be an rPET concentration of 15% (v/v), a CA concentration of 6% (v/v), an electrical voltage of 13 kV, a needle-collector distance of 18 cm, and an injection speed of 0.1 mL/h. The nanofiber structure and morphology were assessed by using attenuated total reflectance-infrared Fourier transform infrared (ATR-FTIR), thermogravimetric analysis (TGA), and scanning electron microscopy (SEM) analyses. Then, PPL was immobilized onto the nanofibers through adsorption and cross-linking methods. The optimum temperature for free PPL was determined to be 30 °C, and the optimum temperature for PPL immobilized on rPET/CA was determined to be 40 °C. It was observed that, especially under acidic conditions, after the immobilization process, PPL immobilized rPET/CA nanofibers became more resistant to pH changes than free PLL. Furthermore, the immobilized PPL exhibited improved pH stability, reusability, and thermal stability compared to its free counterpart. This innovative approach not only contributes to plastic waste reduction but also opens new avenues for enzyme immobilization with potential applications in biocatalysis and wastewater treatment.

## 1 Introduction

Lipases (triacylglycerol hydrolases E.C. 3.1.1.3) are from the family of serine hydrolases produced from different sources, such as microorganisms, vegetables, and animals, and are found in all organisms.^1^ Lipases attract the attention of researchers due to their properties, such as recognition of a wide variety of substrates, enantioselectivity, and high stability.^2^ Lipases are one of the most important enzymes in enzyme technology as they can catalyze many different reactions, such as degradation reactions of long-chain triglycerides, esterification, transesterification, aminolysis, acidolysis, and alcoholysis, due to their wide specificity toward various substrates. For this reason, they are used in many different areas, such as wastewater treatment, the paper industry, diagnostic kits, aroma and biodiesel production, additives in laundry detergents, the textile industry, digestive aids, personal care, and cosmetic products.^3−6^

Lipases differ from other enzymes because they show a unique mechanism of action called interfacial activation.^7,8^ The active center of most lipase molecules is covered by a polypeptide chain called a “cap”, which can isolate the active center from the reaction media in a homogeneous medium (closed form).^9−12^ If there is a hydrophobic surface in the environment, the enzyme adsorbs to this surface, completely exposing the active center and creating a new structure called open, and therefore lipases can hydrolyze the oil drops.^7,8^ Thanks to this view, open forms of many lipases have been selectively used for immobilization on various hydrophobic carriers.^13−17^ The open form of a lipase molecule can form dimers with altered catalytic properties when other lipases stabilize the open form. This allows for the effective use of immobilized lipases to selectively adsorb other lipase molecules.^18−21^ The open form of lipases generally occurs when the lid moves in the presence of hydrophobic surfaces, resulting in an increase in enzyme activity.^22−24^ In addition, inhibition of the activity of pancreatic lipase can be observed in the presence of phenolic compounds such as caffeic acid, capsaicin, quercetin, and *p*-coumaric acid.^25^ These properties can be modulated by physical or chemical modifications, genetic manipulations, or immobilization via interfacial activation on hydrophobic supports.^26−29^

Enzyme immobilization is the fixation of enzyme molecules on or in a solid matrix.^30^ Enzyme immobilization methods can be classified as chemical (cross-linking and covalent bonding) and physical (encapsulation, adsorption, and cross-linking) methods.^31^ Immobilization offers numerous advantages over the use of free enzymes.^29^ These include heightened and sustained stability, enhanced selectivity and specificity, greater resilience against protein denaturation caused by chemical solvents, stability across a range of pH levels and temperatures, an increased enzyme/substrate ratio facilitated by an expanded surface area, as well as simplified recovery and reusability of the enzyme.^32,33^ The immobilization technique endows enzymes with a range of distinctive properties that prove more promising on an industrial scale than their soluble counterparts.^34,35^ The structure of the support material is extremely important for the success of enzyme immobilization and the development of enzymatic properties. Various materials, from natural polymers to expensive synthetic polymers, have been used as solid support porous and nonporous nanomaterials in immobilization.^36,37^ Enzymes immobilized on nonporous nanoparticles offer kinetic advantages over other immobilized biocatalysts; however, it is important to note that the stabilization effects of immobilization on porous solids might diminish or be completely lost. Furthermore, these materials could potentially limit the range of applicable reactions. Nonetheless, for specific substrates, such as solids, they may represent the sole viable alternative.^27^

Particularly, developments in nanotechnology have played an important role in the development of many nanomaterials that can be used in enzyme immobilization.^38^ Among these nanomaterials, nanofibers are more preferred in the immobilization process due to their large surface area, mechanical strength, biocompatibility, and nontoxicity.^39^ Various methods have been used to produce nanofibers. Electrospinning, which is one of these methods, is the most economical method to produce nanofibers with a porous structure, a homogeneous diameter, and a large surface area. Due to these properties, nanofibers produced from natural and synthetic polymers using the electrospinning method have been frequently used as carriers in enzyme immobilization in recent years.^40,41^

The immobilization of lipases aids in enhancing operational stability and facilitates the extraction of the enzyme from the reaction system, allowing for significant reusability in continuous operations. This efficiency in reuse contributes to its cost-effectiveness and amplifies its industrial applications.^42,43^ Recent trends indicate a shift toward reducing the size of support materials, which has been shown to improve the yield of immobilized enzymes. Nanodimensional carriers exhibit the aforementioned desirable characteristics for serving as supporting carriers in lipase immobilization. These traits include high surface area and mechanical strength, along with reduced diffusion limitations, thereby offering a promising avenue for conducting highly efficient biocatalysis procedures.^26,44^ Strategically designing and optimizing enzymatic immobilization on nanofibers have the potential to significantly enhance enzymatic catalytic performance.

Poly(ethylene terephthalate) (PET) is a copolymer of terephthalic acid and ethylene glycol and is a thermoplastic polyester-based polymeric material.^45^ PET is generally used in the production of plastics used in the manufacture of food packaging and beverage bottles.^46^ Approximately 13 million tons of PET are consumed annually in the world, and it is estimated that this value will double in 20 years.^47^ As a result of the increased consumption of PET-based products in recent years, these plastics, which have a long decomposition time, cause serious environmental problems. Unfortunately, only a few of these PET-based products are recycled, and the rest are discarded, raising environmental issues of great concern. For these reasons, it is extremely important to develop new methods for converting PET-based products into more valuable products.^48,49^

PET has been successfully used in the production of recycled PET (rPET) nanofibers by researchers in recent years due to its tensile strength, low cost, biocompatibility, transparency, easy processing, and high nanofiber-forming properties.^50,51^ Despite these advantages, PET nanofibers have limited usage areas due to their hydrophobic structure.^52^ In order to overcome this, it is possible to change the surface properties of PET nanofibers by forming composites with other natural or synthetic polymers during the production of PET nanofibers.^53,54^ CA a derivative of cellulose is a nontoxic, biocompatible, inexpensive, and biodegradable biopolymer. CA, a soluble esterified derivative of cellulose, dissolves in organic solvents relative to cellulose, and as a result, it can be mixed with many compounds and polymers.^55−57^ In addition, due to the easy electrospinning feature of CA, it is possible to obtain nanosized fibers with a large surface area and high porosity that can be used in different applications.^58^

Despite the lack of precise knowledge regarding the specific structure responsible for glutaraldehyde properties, leveraging its versatility can offer certain advantages when utilized effectively.^59^ It has been reported that there are at least 3 ways to immobilize an enzyme using glutaraldehyde.^60,61^ One approach involves treating enzyme molecules that were initially immobilized through ion exchange on aminated supports with glutaraldehyde.^60^ The process should be gentle to guarantee that each primary amino group in both the enzyme and the support undergoes modification with only a single glutaraldehyde molecule.^62^ The amino-glutaraldehyde compound exhibits high reactivity with similar groups but minimal reactivity with free primary amino groups.^63^ However, this process may affect the properties of the enzyme due to the complete replacement of the protein surface with glutaraldehyde.^64^ The remaining two strategies for immobilization involve the use of preactivated supports with glutaraldehyde.^65^ In such scenarios, the glutaraldehyde treatment must be sufficiently intense to ensure that all amino groups acquire two glutaraldehyde molecules, while also averting uncontrolled polymerization of glutaraldehyde.^62^ The amino-glutaraldehyde-glutaraldehyde groups exhibit high reactivity with nonionized primary amino groups but not with other glutaraldehyde molecules.^59,60,62,65^ Enzymes can be immobilized on this support under low ionic strength conditions, initiating a first ion exchange that already immobilizes the enzyme. Subsequently, certain nucleophilic groups of the protein may react with the glutaraldehyde groups on the support, forming covalent attachments.^64^

In this study, glutaraldehyde (GA) was employed due to its bifunctional nature and its efficacy as a potent cross-linker for both enzymes and support materials. GA interacts primarily with the primary amino groups of proteins among various enzyme moieties, although it can eventually react with other functional groups such as thiols, phenols, and imidazoles as well.^66^ The stability and activity of enzymes immobilized on GA-activated supports are contingent on the specific immobilization procedure employed. Different functional groups can indeed lead to variations in stability. A shorter spacer arm (monomer) might offer increased rigidity, potentially enhancing stability, while a longer spacer arm (dimer) could allow for reactions with more groups, potentially optimizing outcomes.^26,67^

In this study, rPET/CA nanofibers were produced by using the electrospinning method. Wastewater bottles were used as the PET source. rPET/CA nanofibers were not only characterized but also used as carrier to explore their potential utility in PPL immobilization, and their performance as a nanobiocatalyst was examined. The morphological and structural properties of these composite nanofibers were determined by using scanning electron microscopy (SEM), FTIR, and thermogravimetric analysis (TGA). PPL was first immobilized on rPET/CA nanofibers using adsorption and then cross-linking methods with GA. In the literature, it has been reported that trypsin, laccase, and lipase were immobilized on PET-based nanofibers.^28,37,68,69^ However, none of these studies employed waste materials as the source of PET for their experiments. This study differs from its counterparts in the literature in that wastewater bottles are used as PET sources in the production of PET-based carrier nanofibers in PPL immobilization.

## 2 Results and Discussion

### 2.1 Fabrication of rPET/CA nanofiber

The production parameters for rPET/CA nanofibers used in PPL immobilization, including rPET concentration, CA concentration, electrical voltage, distance between the needle and collector, and injection speed, were determined and are summarized in Table 1. The selection of the optimal nanofiber structure was based on several criteria, including the formation of a Taylor cone during electrospinning, the absence of polymer droplets at the needle tip or on the collector, system stability, mechanical stability of the fibers, ease of fiber removal from the collector, and overall quality of fiber formation. The optimal production parameters for rPET/CA nanofibers used for PPL immobilization were found to be an rPET concentration of 15% (w/v), a CA concentration of 6% (w/v), an electrical voltage of 13 kV, a needle-collector distance of 18 cm, and an injection speed of 0.1 mL/h.

**Table 1:** Observation and Operational Parameters of rPET/CA Nanofibers

PET concentration (% w/v)	CA concentration (% w/v)	voltage (kV)	needle-collector distance (cm)	injection speed (mL/h)	observation^a^
10	5	11	16	0.3	-
10	5	13	16	0.3	-
10	5	15	16	0.3	-
10	6	13	18	0.1	+
10	6	15	18	0.1	++
10	7	15	20	0.3	+
15	5	13	16	0.1	-
15	6	13	18	0.1	+++
15	6	15	16	0.3	++
15	7	15	20	0.1	+
20	5	13	16	0.1	+
20	5	15	18	0.3	+
20	6	13	16	0.1	++
20	6	15	18	0.3	+
20	7	11	16	0.1	-
20	7	13	18	0.3	+
20	7	15	20	0.1	++

a Criteria of positive (+) observation: The Taylor cone forms during electrospinning, and there are no polymer droplets to develop at the needle tip or on the collector. The system is stable, and the fiber may be removed from the collector with ease.

### 2.2 Characterization of the rPET/CA Nanofibers

#### 2.2.1 SEM Analysis

**Figure 1:**
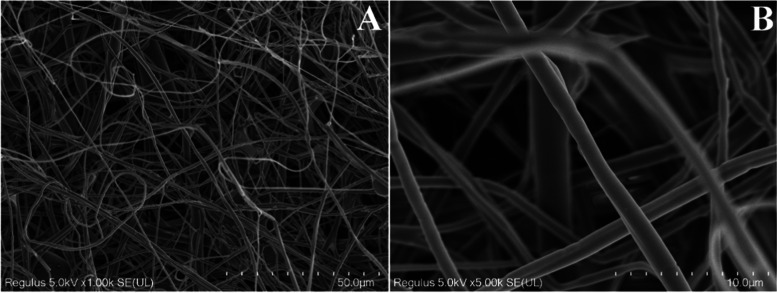
SEM images of rPET/CA nanofibers at various magnifications (A) 1000× and (B) 5000×.

**Figure 2:**
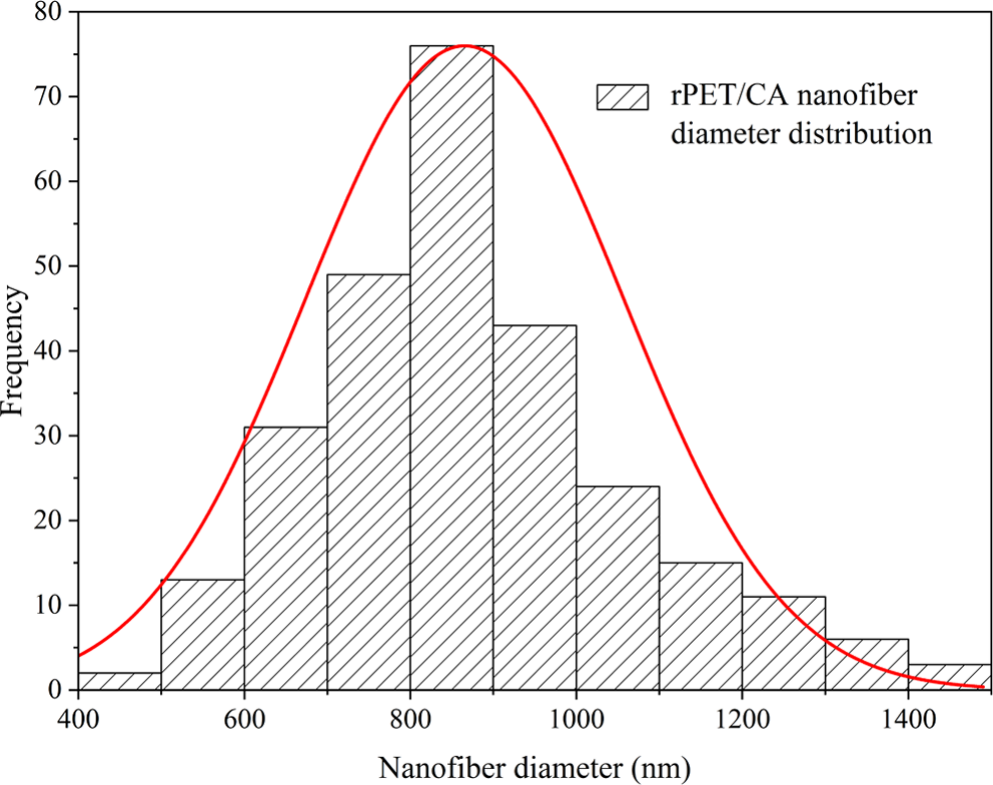
Size distribution of the rPET/CA nanofiber.

**Figure 3:**
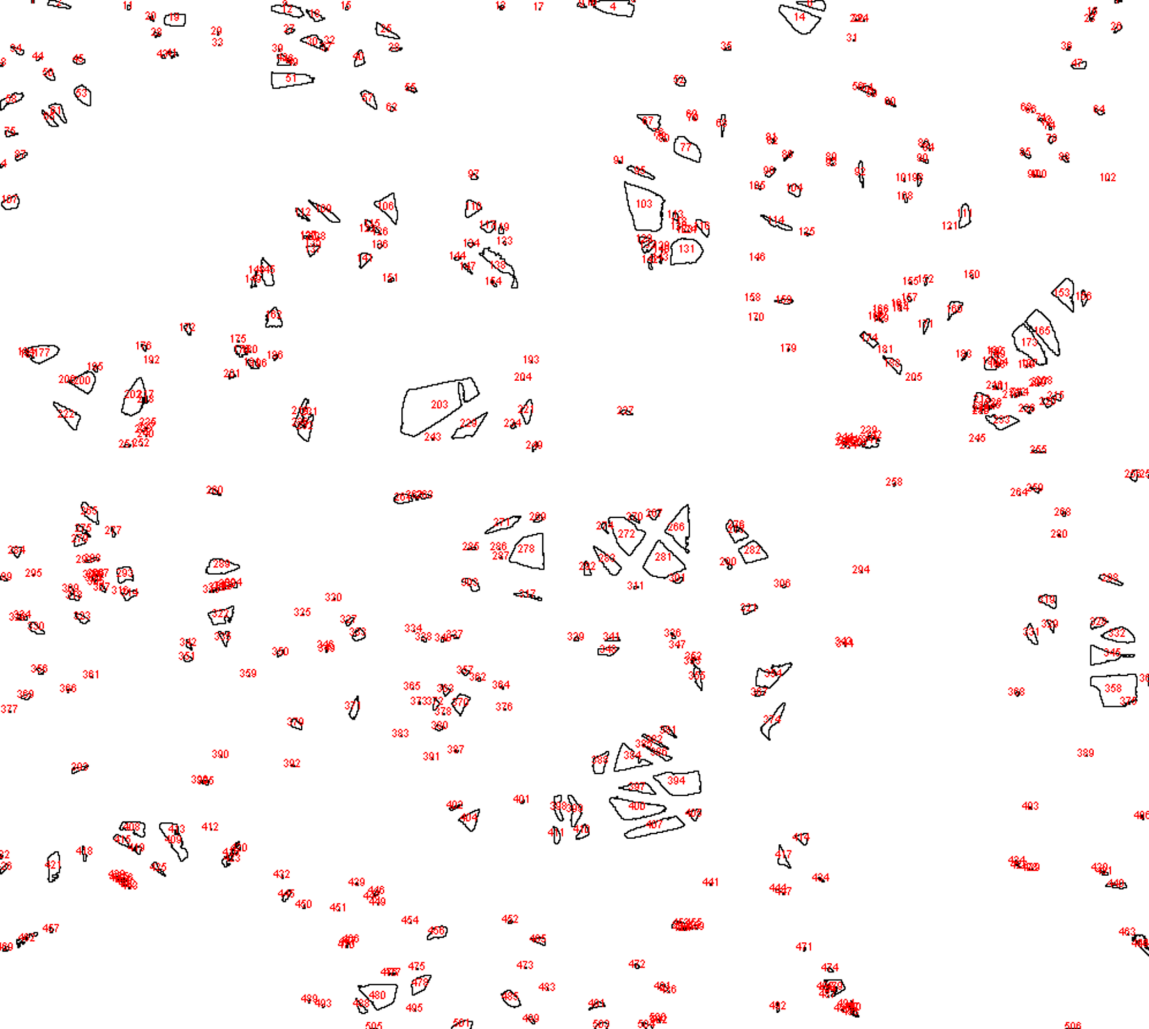
After image analysis rPET/CA nanofiber.

**Figure 4:**
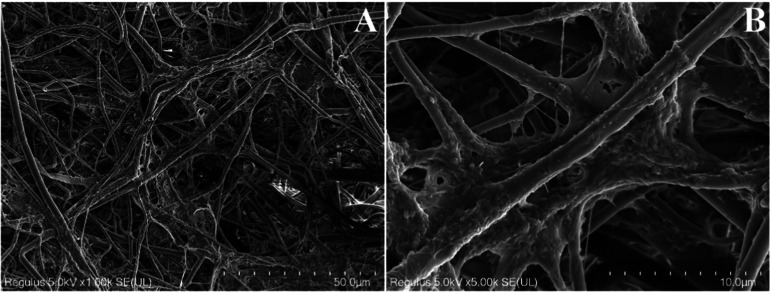
SEM images of PPL immobilized rPET/CA nanofibers at various magnifications (A) 1000× and (B) 5000×.

Scanning electron microscopy (SEM) is a powerful imaging technique used in the field of microscopy to visualize the surface and topographical features of a wide range of materials at the microscopic and nanoscopic scale. It works by scanning a focused electron beam over the sample’s surface and collecting the electrons that are emitted as a result of this interaction. SEM provides high-resolution three-dimensional (3D) images of the sample, allowing for a detailed examination of its structure, morphology, and surface characteristics. The SEM technique was used to determine the morphology of the rPET/CA nanofibers produced by the electrospinning method before and after PPL immobilization. As seen in Figure 1, the rPET/CA nanofibers exhibited a structure characterized by nonporous, randomly arranged, homogeneous, smooth, and free-of-beads fibers. Certainly, the substantial surface area of these acquired nanofibers holds significant importance, especially for facilitating enzymatic reactions and the immobilization of enzymes. Furthermore, the diameters of the rPET/CA nanofibers were calculated to be approximately 860 nm, and the size distribution of these fibers is illustrated in Figure 2. Additionally, Figure 3 shows the image used to determine the surface area and porosity by numbering each space on the rPET/CA nanofiber. The surface area and porosity of the rPET/CA nanofibers were determined to be 888.432 μm and 29.445%, respectively, employing image analysis conducted by using ImageJ software. It was determined that there were changes in the SEM images of rPET/CA nanofibers after PPL immobilization (Figure 4). Observations revealed that the nanofiber surfaces were notably coated with fine particles, resulting in an increased surface roughness. While the overall fiber integrity remained intact, it was observed that the gaps between the fibers were filled by enzyme molecules. These findings support the successful immobilization of PPL onto the rPET/CA nanofibers.

#### 2.2.2 FTIR Analysis

**Figure 5:**
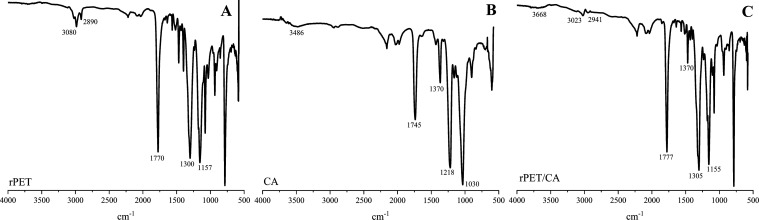
ATR-FTIR spectra of (A) rPET, (B) CA, and (C) rPET/CA nanofiber.

The attenuated total reflectance-infrared Fourier transform infrared (ATR-FTIR) spectra of raw rPET, raw CA, and rPET/CA nanofibers are listed in Figure 5. The distinct absorption bands observed at 3080–2890 cm^–1^ in the raw rPET sample are due to C–H aliphatic and aromatic bond tensions.^70^ The prominent peaks in raw rPET at 1770 and 1300 cm^–1^ can be attributed to the stretching vibrations of the C=O bond and the ester group, respectively.^71^ The band at 1157 cm^–1^ can be associated with the methylene group in the ethylene glycol part of the rPET polymeric segment.^72^ The complex and multiple absorption bands found within the range of 1400 to 800 cm^–1^ may be attributed to phenomena such as geometric isomerization (cis/trans) or distinctions between crystalline or amorphous regions within both phenylene carbonyl and ethylene glycol bonds.^73^ In the CA spectrum, the bands at approximately 3485 and 1745 cm^–1^ signify the presence of −OH and −COOH groups, respectively.^74,75^ The band observed at 1370 cm^–1^ can be attributed to bending vibrations resulting from CH_3_ deformation within the acetate substituent groups. Furthermore, the bands at 1218 and 1030 cm^–1^ correspond to C–O–C vibration stretching and C–O stretching, respectively.^76^ Upon inspecting the spectra of rPET/CA nanofibers, it is evident that some changes have occurred in comparison to the peaks of raw rPET and raw CA. As seen in the ATR-FTIR spectrum of the rPET/CA nanofiber, it was observed that the characteristic peaks of both polymers were preserved, but there were some changes in the intensity of the peaks due to interactions between the functional groups in rPET and CA polymers. Changes were also observed in C–H absorption bands resulting from aliphatic and aromatic bond tensions in the rPET. In addition, slight shifts and changes in the intensity of the vibration peaks resulting from the C=O bond and ester group tensions in raw rPET were observed.

It can be suggested that the −OH functional group of CA and the oxygen atoms in the ester and −O–H groups of PET engage in polar or hydrogen bond interactions. Furthermore, it can be inferred that weak London dispersion or van der Waals interactions formed between the groups on the rPET and CA.

#### 2.2.3 TGA

**Figure 6:**
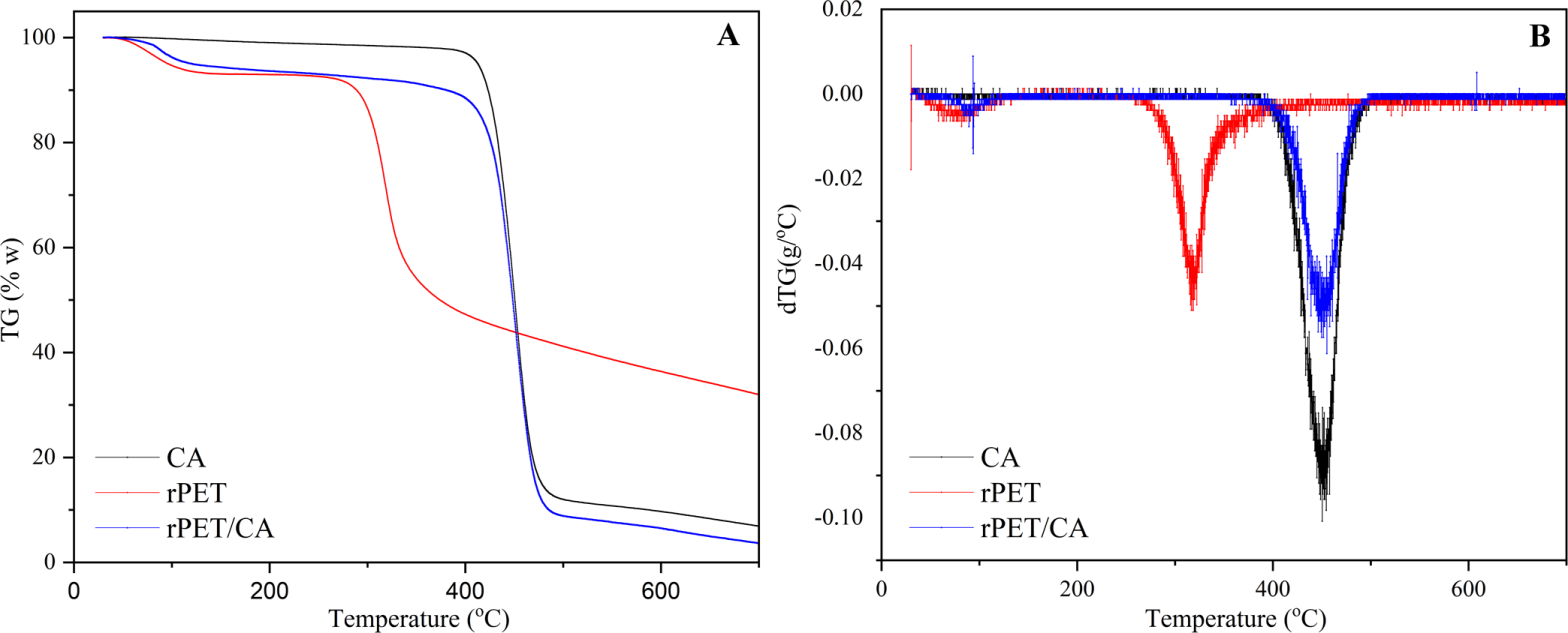
(A) TG and (B) DTG curves of rPET, CA, and rPET/CA nanofiber.

Thermogravimetric (TG) and differential thermogravimetric (DTG) curves for rPET, CA, and rPET/CA nanofibers are depicted in Figure 6. Upon examination of the TG and DTG curves derived from thermal gravimetric analysis of rPET, it was evident that the degradation occurred in four stages. These four stages likely correspond to the decomposition of various compounds within the rPET structure as it undergoes thermal degradation. This result is consistent with previous studies.^77,78^ In the first stage, the decrease of approximately 6% below 120 °C can be interpreted as resulting from the loss of CO and CO_2_ in the rPET structure. A slight decrease was observed between 120 and 270 °C, and at the second stage, 3% of the rPET mass was seen to move away from the structure. The decrease in mass observed at this stage may be due to the loss of C_2_H_6_O_2_ and C_2_H_4_O groups in the structure. In the third stage, approximately 40% of the rPET mass moved away from the structure, with a sharp decrease between 270 and 335 °C. The mass loss observed at this stage may be due to the removal of C_4_O_2_. In the last step, observed between 335 and 450 °C, it can be said that approximately 10% of the mass loss is caused by the RCO-OR groups present in the structure. According to the DTG curve of rPET, the maximum rate of degradation occurred at 320 °C.

According to the TG and DTG curves obtained by the thermal gravimetric analysis of CA, it was seen that thermal degradation occurred in three stages. In the first step, the 3% mass decrease observed in the CA structure below 390 °C may be due to the removal of moisture or volatile substances in the structure. In the second stage, which is the main degradation step, it was observed that 88% of the mass was removed from the structure between 390 and 480 °C. It was stated in previous studies that the mass loss occurring in this step was due to the main thermal degradation reactions of CA chains.^79,80^ In the last step, observed between 480 and 700 °C, approximately 93% of the initial mass was observed to move away from the structure. Additionally, it was determined that the maximum decomposition temperature of CA was 450 °C from the DTG curve.

When the TG and DTG curves of the rPET/CA nanofiber were examined, it was seen that the rPET and CA in the structure showed differences according to the TG and DTG curves. Different thermal decomposition curves may have been observed due to the interaction of the groups on rPET and CA. A 7% decrease in mass was observed in the rPET/CA nanofiber up to 140 °C. The mass decrease that occurs at this stage may be due to the removal of moisture, CO, and CO_2_ from the structure. In the second stage, it was observed that approximately 4% of the mass moved away from the structure between 190 and 390 °C. The reason for this decrease may be due to the complete removal of C_2_H_6_O_2_ and C_2_H_4_O and the partial removal of C_4_O_2_ in the structure of rPET in the nanofiber structure. The primary decomposition step, occurring between 310 and 480 °C, represents the third stage, during which approximately 90% of the initial mass was decomposed. It can be said that the mass loss observed at this stage is due to the main thermal degradation reactions of the rPET and CA chains. The last step took place between 480 and 700 °C, and at the end of the analysis, the ash residue amount of the rPET/CA nanofiber was found to be 3.5%. The maximum degradation temperature of the rPET/CA nanofiber was found to be 450 °C. Based on these TGA results, it can be inferred that the presence of CA in the structure enhances the thermal stability of the nanofiber, likely due to interactions with rPET. According to this result, it can be inferred that rPET/CA nanofibers have the potential to enhance the thermal stability properties of enzymes following the immobilization process.

### 2.3 Optimization Studies of PPL Immobilization on rPET/CA Nanofiber

Biocatalysis is integral to green and sustainable chemical manufacturing. It offers a pathway to cleaner, more efficient, and environmentally friendly processes. Immobilization of enzymes is a key enabling technology that enhances the practical and commercial viability of biocatalysis. It addresses the challenges associated with free enzymes and unlocks the full potential of enzymatic catalysis in sustainable chemical manufacturing. As a result, the combination of biocatalysis and immobilization holds great promise for a more sustainable and environmentally responsible future in the chemical industry.

**Figure 7:**
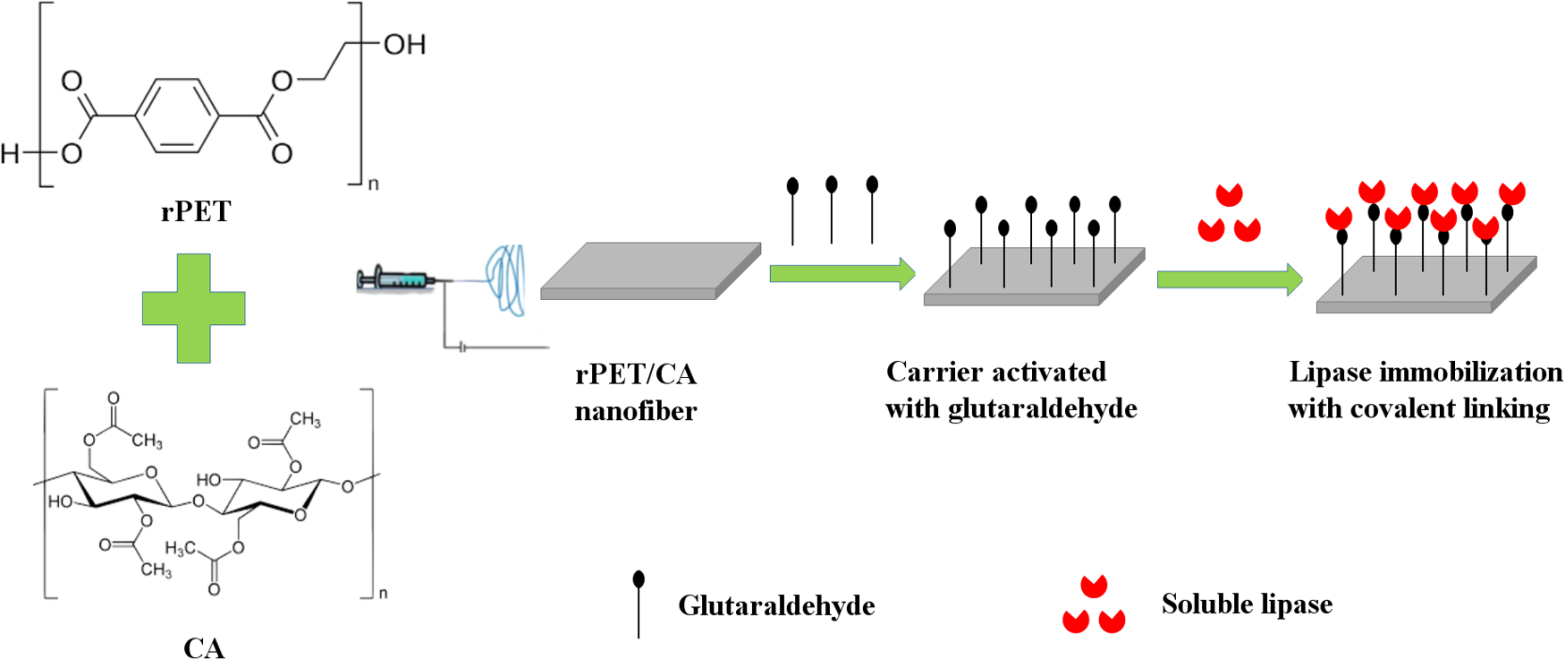
Schematic representation of the fabrication of rPET/CA and PPL immobilization on a glutaraldehyde-activated carrier.

**Figure 8:**
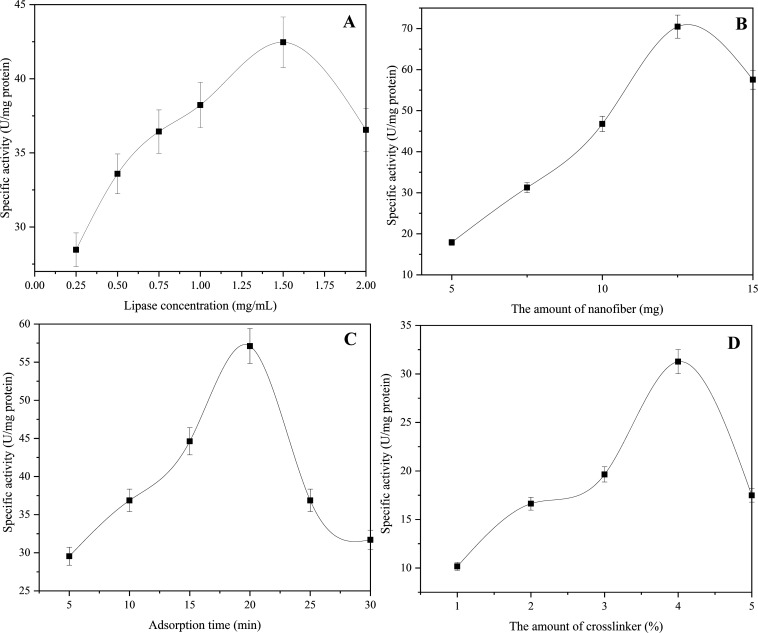
Optimization studies results of PPL immobilization on rPET/CA nanofiber (A) PPL concentration, (B) nanofiber amount, (C) adsorption time, and (D) GA amount.

In this study, first, rPET/CA nanofibers were produced by the electrospinning method, and then the PPL enzyme was immobilized on these nanofibers activated with GA (Figure 7). The choice of spacer arm is crucial, as it can impact steric hindrances, accessibility of the enzyme’s active site, and overall immobilization efficiency. The spacer arm should be carefully selected to balance these factors for optimal enzyme immobilization. Glutaraldehyde is a bifunctional cross-linking agent that can react with both the enzyme and the support material, forming covalent bonds. Therefore, covalent linkage using glutaraldehyde as a cross-linking agent is an effective method for enzyme immobilization due to its stability, reusability, and control over the immobilization process. In optimization studies of PPL immobilization on rPET/CA nanofiber, parameters such as PPL amount, nanofiber amount, cross-linker amount, and adsorption time were investigated, and the results are displayed in Figure 8.

In order to investigate the effect of PPL concentration on the optimization of immobilization, PPL solutions ranging from 0.25 to 2.5 mg/mL were immobilized on 10 mg of carriers. Other optimization parameters were kept constant during optimal PPL concentration experiments (3% cross-linker amount and 15 min adsorption time). It was observed that the activity gradually increased as the PPL concentration increased, but the activity decreased after the concentration of 1.5 mg/mL, which is the optimum amount of PPL (Figure 8A). The reason for the decrease in activity at concentrations above the optimum PPL concentration may be that the amount of rPET/CA nanofibers is not sufficient for PPL immobilization. Additionally, it may be a result of multiple adsorptions of PPL molecules onto the nanofiber surface.

İspirli et al. reported the optimum lipase concentration as 1.5 and 1 mg/mL for PEO/AL and PVA/AL nanofibers, respectively.^81^ In the study conducted by Li et al., the optimum lipase concentration was determined to be 5 mg/mL for polyacrylonitrile nanofiber.^82^ In another study, this value was found to be 10 mg/mL for polysulfone nanofiber.^83^ The optimum amount of lipase needed for immobilization can vary depending on the type of carrier material used. Different carrier materials have varying capacities to bind and support enzymes; therefore, the ideal enzyme concentration for effective immobilization may differ from one carrier to another.

To determine the optimum carrier amount, 1 mL of PPL solution (1.5 mg/mL) was immobilized on nanofibers varying between 5 and 15 mg, and the results are given in Figure 8B. Meanwhile, other optimization parameters, such as the amount of cross-linker (3%) and adsorption time (15 min), were kept constant. The optimum amount of nanofiber for immobilization of PPL on rPET/CA nanofiber was found to be 12.5 mg. When the nanofiber amount is below the optimum level, low enzyme activity might be observed because there are not enough carriers available to bind with the enzyme molecules effectively. Conversely, when the nanofiber amount exceeds the optimum, low enzyme activity could also occur. This is because an excess of carriers in the environment may sterically hinder the interaction between the enzyme and the substrate molecules, impeding the enzymatic reactions. In a study in the literature, it was reported that the optimum amount of nanofiber for lipase immobilization was 5 mg.^84^ In another study, it was determined that the optimum carrier amount was 20 mg for glutaraldehyde-activated poly(vinyl alcohol-*co*-ethylene) nanofibers.^85^

One mL portion of PPL solution (1.5 mg/mL) was immobilized on 12.5 mg of rPET/CA nanofiber for 5 to 30 min to find the optimum adsorption time, and the results are presented in Figure 8C. The amount of cross-linker was kept constant (3%) throughout the optimum adsorption time studies. At the conclusion of the 20 min duration, it was noted that the surface of the rPET/CA nanofibers had become saturated with PPL molecules. A decrease in specific activity may have been observed as PPL molecules began to desorb from the rPET/CA nanofiber surface at times above the optimum adsorption time. İspirli Doğaç et al. reported the adsorption time for the immobilization of lipase to PEO/AL and PVA/AL nanofibers as 20 min.^81^

To determine the optimum cross-linker amount, 1 mL of PPL solution (1.5 mg/mL) was immobilized on 12.5 mg of rPET/CA nanofibers using GA solutions ranging from 1 to 5% for a 20 min adsorption period, and the results are shown in Figure 8D. The optimal concentration of the cross-linker was determined to be 4%. When the amount of cross-linking agent in the medium falls below this value, it may not provide sufficient cross-linking for the enzyme molecules to immobilize effectively on the carrier. Conversely, when the amount exceeds the optimum level, it could potentially lead to denaturation of the enzyme molecules due to an excessive concentration of the cross-linking agent. In the immobilization studies of lipase on Zr-MOF/PVP nanofiber, the optimum amount of GA was found to be 2.5%.^86^ The optimum amount of GA for laccase enzyme immobilization on PET-based nanofiber was found to be 0.45%.^37^ In another study, it was stated that the optimum GA value for trypsin immobilization on PET-based nanofiber was 0.05%.^28^

The activity properties of free PPL and PPL immobilized rPET/CA nanofibers are given in Table 2. The specific activity of the PPL immobilized rPET nanofiber measured 64.59 U/mg protein, with a protein content of 0.34 mg, while the free PPL exhibited values of 69.88 U/mg protein and 0.65 mg, respectively. After the immobilization, there was a reduction in protein content by approximately 58%, whereas the specific activity yield increased by around 86.33%. It can be said that almost the majority of the active enzyme molecules in the immobilized free PPL are immobilized on the rPET/CA nanofiber. In addition, it can be said that activating GA before the immobilization of rPET/CA nanofibers contributes to the immobilization of PPL.

**Table 2:** Activity Properties of Free PPL and PPL Immobilized rPET/CA Nanofiber

	activity (U)	protein (mg)	specific activity (U/mg)	bound protein (%)	specific activity yield (%)
free PPL	45.42	0.65	69.88	-	100
immobilized PPL	21.96	0.34	64.59	52.31	92.43

### 2.4 Characterization Studies of Free PPL and PPL Immobilized rPET/CA Nanofibers

Lipase activity and stability are crucial attributes in biocatalytic applications due to their fundamental roles in facilitating enzymatic reactions. These characteristics determine the efficiency and practicality of using lipases as biocatalysts. In our study, properties such as the effect of temperature, the effect of pH, reusability, thermal stability, pH stability, and storage stability were tested to evaluate the effectiveness of immobilized PPL on nanofibers for potential biocatalytic applications.

#### 2.4.1 Temperature Properties

**Figure 9:**
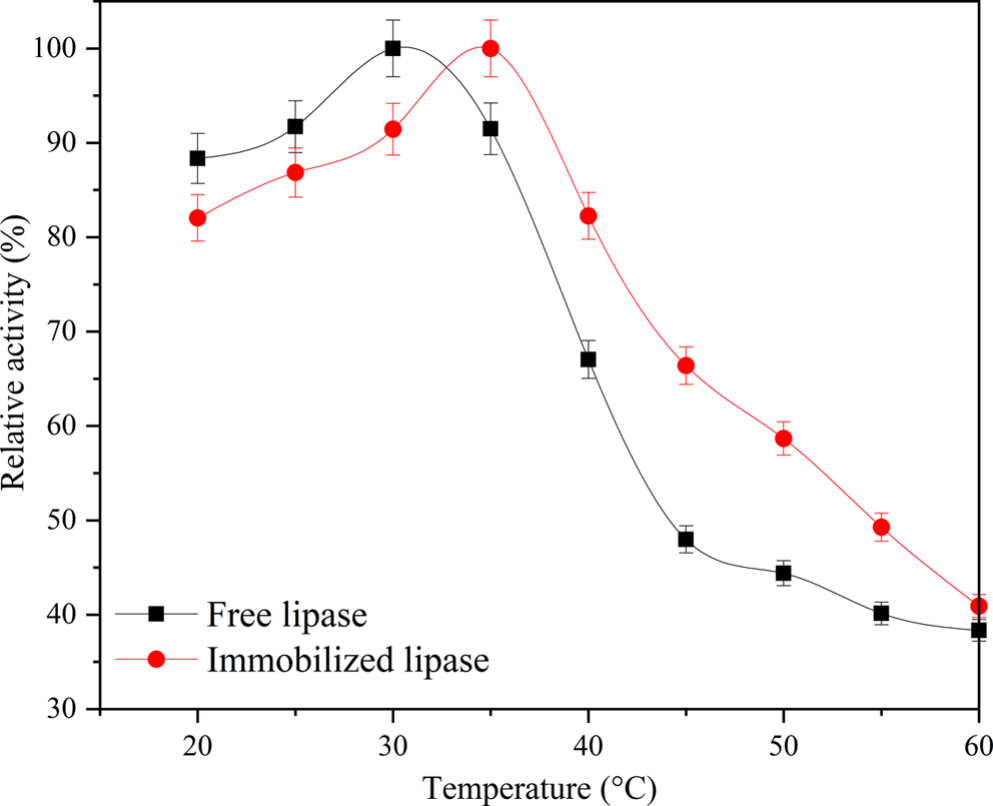
Optimum temperature properties of free PPL and PPL immobilized rPET/CA nanofiber.

To investigate and compare the influence of temperature variation on the activities of both free PPL and PPL immobilized on rPET/CA nanofibers, we conducted activity measurements across a temperature range from 20 to 60 °C. The results of these experiments are illustrated in Figure 9. The optimal temperature for free PPL was determined to be 30 °C, while the optimal temperature for PPL immobilized on rPET/CA was found to be 35 °C. After immobilization, the conformational flexibility of PPL was affected. It can be said that the immobilization of the PPL enzyme on rPET/CA nanofiber increases the rigidity of the PPL, and a higher activation energy is required compared to free PPL to rearrange the immobilized PPL in the appropriate conformation for the formation of the enzyme substrate complex. Furthermore, it was observed that immobilized PPL exhibited higher activity than free PPL across all temperature values studied above the optimum temperature for immobilized PPL. Immobilizing a free enzyme onto a carrier can indeed lead to several beneficial effects, including increased rigidity of the enzyme structure and protection against denaturation caused by heat. These factors can collectively result in the immobilized enzyme having a higher optimum temperature compared to its free counterpart. Consequently, the higher enzyme activity observed at elevated temperatures can be attributed to these advantageous effects of immobilization. Similar studies have reported an increase in the optimum temperature value following the immobilization process. In the study conducted by Huang et al., as a result of lipase immobilization on cellulose nanofibers, the optimum temperature value for immobilized lipase was found to be 40 °C, while this value was 35 °C for free lipase.^84^ Lipase enzyme was immobilized on UiO-66/PVDF nanofiber, and the optimum temperature values were found to be 30 and 50 °C for free lipase and immobilized lipase, respectively.^87^

**Figure 10:**
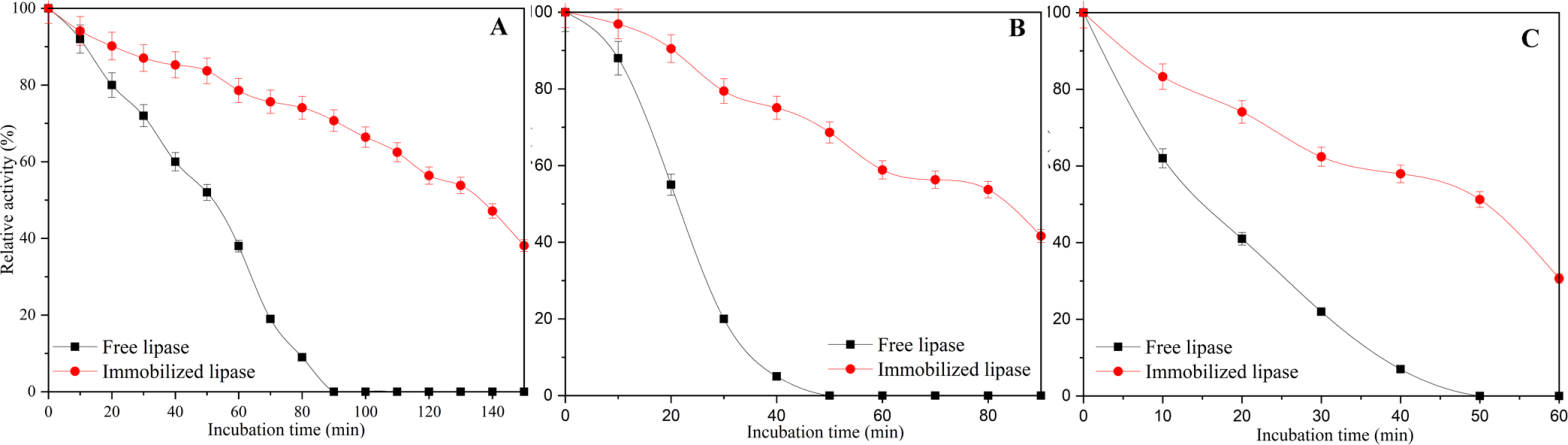
Thermal properties of free PPL and PPL immobilized rPET/CA nanofiber at (A) 50 °C, (B) 60 °C, and (C) 70 °C.

The results of thermal stability studies performed at 50, 60, and 70 °C for free PPL and the PPL immobilized rPET/CA nanofiber are given in Figure 10. At the end of 80 min at 50 °C, free PPL activity lost almost all of its activity, while PPL immobilized rPET/CA nanofiber retained approximately 74% of its activity. The free enzyme lost almost all of its activity after 40 min at 60 °C and after 20 min at 70 °C. At the same temperature values and at the same time, PPL immobilized rPET/CA nanofibers managed to preserve approximately 75% of their activity. This result can be attributed to the protective environment provided by rPET/CA nanofiber, which shields the enzyme from denaturation and other detrimental effects caused by the elevated temperature. Additionally, the immobilization process may have increased the overall stability of the enzyme, allowing it to maintain its functionality under such conditions. These findings highlight the practical advantages of using immobilized enzymes in various applications, especially when the enzymes need to operate under challenging conditions, such as high temperatures.

#### 2.4.2 pH Properties

**Figure 11:**
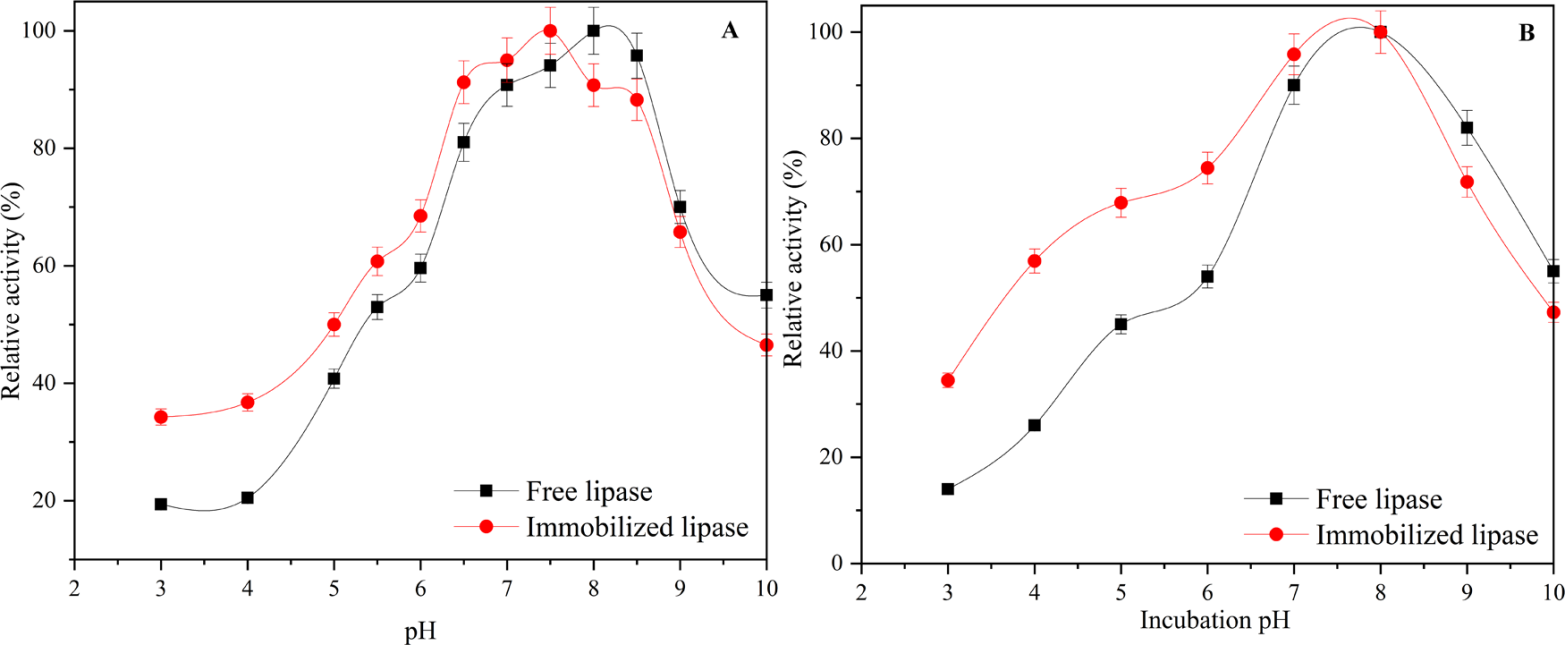
(A) Optimum pH profiles of free PPL and PPL immobilized rPET/CA nanofiber; (B) pH stabilities of PPL and PPL immobilized rPET/CA nanofiber.

To investigate the effect of pH on the activities of free PPL and PPL immobilized rPET/CA nanofibers, activity measurements were made at pH values ranging from 3 to 10, and the findings are presented in Figure 11A. The optimum pH value for free PPL was found to be pH 8, while the optimum pH value for PPL immobilized on rPET/CA was found to be pH 7.5. The protective microenvironment provided by the negatively charged CA surfaces in the nanofiber structures, combined with the stabilizing effects of immobilization, likely contributes to the observed shift in optimal pH and increased activity under acidic conditions for the PPL immobilized on rPET/CA nanofibers. This improved enzyme performance of immobilized PPL on a wider pH range allows it to be used on a pH scale wider than that of the free enzyme. There are some studies in the literature that show a change in the optimum pH value after the immobilization process.^86,88,89^

The results of pH stability studies conducted for free PPL and PPL immobilized rPET/CA nanofibers are given in Figure 11B. After the immobilization process, it was observed that the pH stability properties of the PPL enzyme improved, especially in the acidic region. It can be said that the immobilization of PPL on rPET/CA nanofiber results in increased pH stability, especially in acidic conditions, due to the protective microenvironment created by the carrier, the pH buffering properties of the nanofiber, and the reduced sensitivity of the immobilized enzyme to pH fluctuations.

### 2.5 Reusability and Storage Capacity

**Figure 12:**
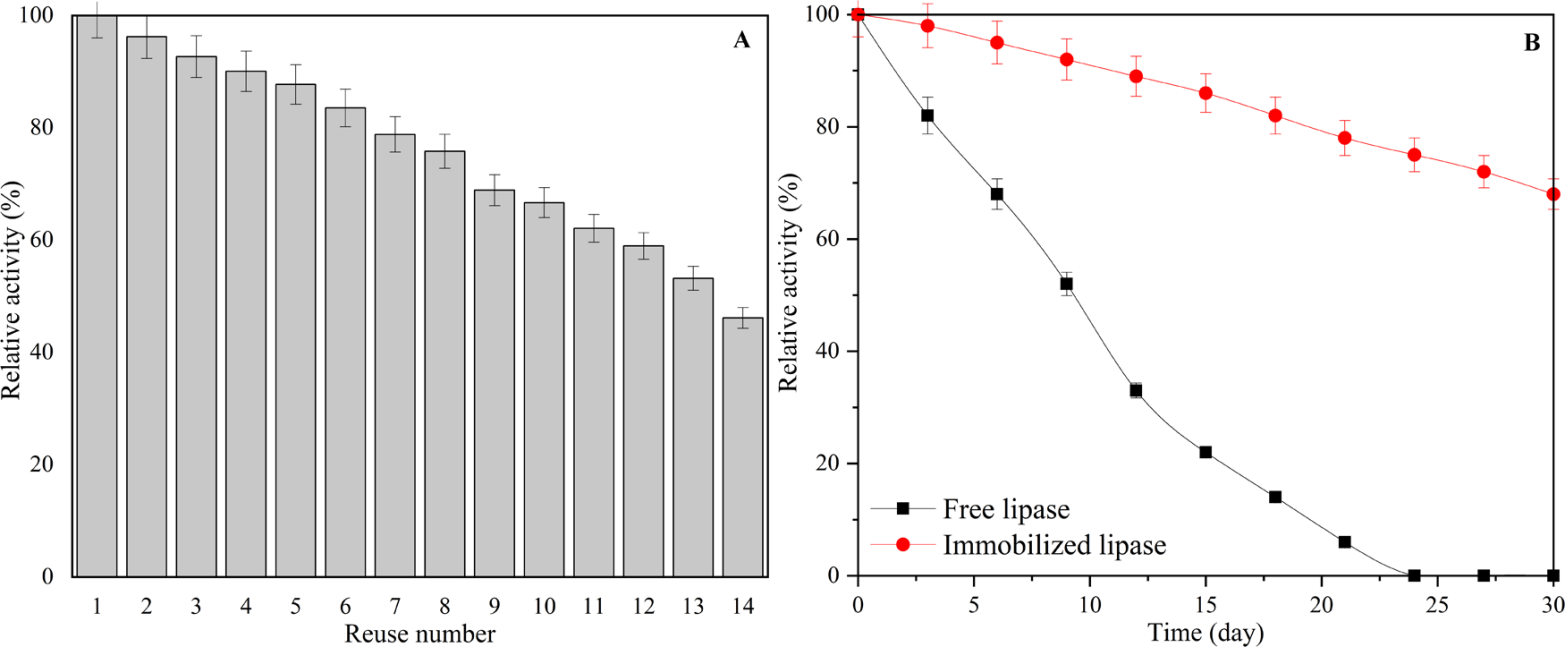
(A) Reuse number of PPL immobilized rPET/CA nanofiber and (B) the storage stability of free PPL and PPL immobilized rPET/CA nanofiber.

One of the primary advantages of enzyme immobilization is its ability to facilitate enzyme reuse, contributing to its cost-effectiveness and sustainability in various applications. To reveal the reuse performance of PPL immobilized rPET/CA nanofiber, PPL activity was measured repeatedly under optimum conditions (Figure 12A). The observed decrease in PPL activity after multiple reuses of the immobilized rPET/CA nanofiber can be attributed to several factors. Initially, during repeated use, there might be a gradual loss of enzyme molecules from the nanofiber due to mechanical forces, desorption, or other factors. Additionally, over time, some structural changes or damage might occur within the immobilized enzyme or the carrier material, affecting the overall enzyme activity. It is also possible that the repeated exposure to the substrate and reaction conditions leads to some irreversible changes in the enzyme’s active sites. Despite this gradual decline in activity, the immobilized PPL still demonstrates significant reusability, retaining more than 50% of its activity after 13 uses, making it a valuable option for various applications in which enzyme reusability is crucial.

The storage capacity of the immobilized enzyme is a critical parameter that reflects the effectiveness of the immobilization process and holds significant importance in biocatalytic reactions. The storage stability of both free PPL and PPL immobilized rPET/CA nanofibers was assessed by monitoring their activity every 3 days at 25 °C over a period of 30 days (Figure 12B). After 12 days of storage, free PPL experienced a significant decrease in activity, retaining only 35% of its initial activity, whereas PPL immobilized rPET/CA showed remarkable stability, retaining 89% of its activity during the same period. Furthermore, while free PPL lost nearly all of its activity after 21 days of storage, the immobilized PPL maintained approximately 70% of its activity even after 30 days of storage, highlighting the superior storage stability of the immobilized enzyme. The observed loss of activity in free PPL over time, which is attributed to autolysis, is a natural enzymatic process where the enzyme molecules start to degrade themselves under certain conditions, leading to a decrease in their catalytic activity.^90,91^ In contrast, PPL immobilized on rPET/CA nanofibers is less susceptible to autolysis due to the protective microenvironment provided by the carrier, which helps maintain the enzyme’s structural integrity and activity over an extended period. This difference in the autolysis susceptibility further demonstrates the advantages of enzyme immobilization for enhancing enzyme stability and longevity.

### 2.6 Kinetic Parameters

To reveal and compare the kinetic properties of free PPL and the PPL immobilized rPET/CA nanofiber, activity measurements were carried out using different concentrations of *p*-nitrophenyl palmitate solutions. The Lineweaver–Burk plot was used to determine the kinetic parameters of free and immobilized PPL. The *K*_m_ value for free PPL was found to be 0.14 ± 0.02 mM and the *V*_max_ value was 0.42 ± 0.08 U/mg protein; the same values were calculated as 0.17 ± 0.03 mM and 0.35 ± 0.05 U/mg protein for PPL immobilized rPET/CA nanofiber, respectively. The *K*_m_ values can be thought of as the enzyme’s affinity for the substrate. After the immobilization process, there was a slight decrease in the *K*_m_ value. In this case, the fact that *K*_m_ values were not significantly affected after the immobilization procedure suggests that the substrate affinity of PPL has been well preserved. This preservation of substrate affinity can likely be attributed to the structural stability of the PPL enzyme, which is crucial for maintaining its catalytic efficiency, even after immobilization. The immobilization method used in this case seems to have successfully retained the essential structural features of the enzyme, allowing it to interact effectively with the substrate. This is an essential outcome, as it implies that while some properties of the enzyme may change during immobilization, its fundamental catalytic capabilities remain intact. Additionally, the observed decrease in the *V*_max_ value after the immobilization process can be interpreted as the fact that the substrate has a harder time reaching the active site of the immobilized enzyme as a result of the change in the enzyme to the substrate.

## 3 Experimental Section

### 3.1 Materials

Trifluoroacetic acid (TFA), dichloromethane (DCM), cellulose acetate (Mw = 50,000 Da), lipase (from porcine pancreas), triton X-100, glutaraldehyde (GA), *p*-nitrophenyl palmitate (pNPP), and all other chemicals were obtained from Sigma-Aldrich. In order to identify plastic materials as recyclable, they are coded with different code numbers. PET bottles marked with Code-1 indicate that only PET is used without mixing with any other polymer. For this reason, waste pet water bottles with recyclability code 1 were used as a source of PET in this study. PET wastewater bottles were cut into small pieces and cleaned with detergent. Then, the sample was washed with normal water and distilled water, respectively, and left to dry at room temperature.

### 3.2 Fabrication of rPET/CA Nanofibers

A mixture of DCM/TFA (3:1) was used as the solvent system to prepare the rPET/CA polymer solutions. rPET/CA polymer solutions were prepared by using different concentrations of PET (10, 15, and 20%) and CA (5, 6, and 7%). First, PET was stirred into the solvent mixture until completely dissolved. Then, CA was added to this mixture and stirred for 6 h. All operations were carried out at constant room temperature. The polymer solution was inserted into the syringe. The electrospinning system (Inovenso nanospinner) was equipped with a syringe pump (New Era Pump Systems, Inc.). The electrospinning system parameters were determined as flow rate (0.1, 0.3 mL/h), needle tip–collector distance (16, 18, 20 cm), and voltage (11, 13, and 15 kV).

### 3.3 Characterization of rPET/CA Nanofibers

Fourier transform infrared spectroscopy (FTIR) (Thermo Scientific Nicolet iS-5ATR/FTIR Spectrometer) was used to study the chemical structure and surface groups of raw polymers and the rPET/CA nanofiber. Both the raw polymer and rPET/CA nanofiber were subjected to thermal analysis using the PerkinElmer Thermal Gravimetric Analyzer (TGA) 4000 under an N_2_ atmosphere between 50 and 700 °C. PPL was immobilized to rPET/CA nanofibers under optimum conditions (1.5 mg/mL PPL solution, 12.5 mg of rPET/CA, 20 min, 4% (v/v) GA). rPET/CA nanofibers and PPL immobilized rPET/CA nanofibers were coated by sputtering gold at 15 mA for 1 min. Then, SEM images were taken at different magnifications by using a scanning electron microscope (JEOL JSM 7600F). ImageJ software was used to determine the fiber diameters. The average fiber diameter and distribution during measurement were determined by randomly and manually measuring 300 fibers by using representative micrographs. Histogram data of fiber diameters and average fiber diameters were obtained with the help of Origin Pro 2019 software. Additionally, using scanning electron microscope images, surface porosity and surface area values were calculated for rPET/CA nanofibers using ImageJ software.^92^ When making these calculations, the following equation was used:
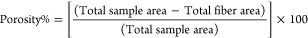


### 3.4 PPL Immobilization on rPET/CA Nanofibers

PPL was immobilized onto rPET/CA nanofibers through adsorption, followed by cross-linking. The optimal conditions for the immobilization of PPL on rPET/CA nanofibers were determined by investigating the parameters of nanofiber amount (5, 7.5, 10, 12.5, and 15 mg), PPL concentration (0.5, 0.75, 1.0, 1.5, and 2.0 mg/mL), adsorption time (5, 10, 15, 20, and 25 min), and amount of cross-linking agent (1, 2, 3, 4, 5%). A known amount of rPET/CA nanofiber was added to 1 mL of a PPL solution of a known concentration, and adsorption was carried out for a certain period of time. Then, a certain amount of cross-linker was added, and cross-linking was carried out for a certain period of time. The nanofiber was separated from the solution and then washed multiple times using distilled water. In this way, the immobilization of PPL on rPET/CA nanofiber was achieved by using the adsorption method followed by cross-linking. According to the optimization results of PPL immobilization, the optimum amount of PPL was found to be 1.5 mg/mL. For this purpose, the PPL enzyme to be used in immobilization was prepared as follows: 15 mg of PPL was taken and dissolved in 10 mL of 50 mM Tris-HCl (pH 8.0) buffer at 25 °C. It was determined that the prepared enzyme solution contained 0.56 mg of protein per mL by the Bradford method. Then, 1 mL of enzyme solution was immobilized on 12.5 mg of rPET/CA nanofiber using 4% GA for 20 min (44.8 mg protein/g nanofiber).

### 3.5 Determination of PPL Activity Assay and Protein Amount

The PPL activity was determined by using the pNPP method. To prepare the substrate solution, first, 9 mL of 50 mM Tris-HCl (pH 8.0) was added to a test tube. Then, 10 mg of gum arabic was added to the tube and mixed by using a vortex until a homogeneous mixture was obtained. The two prepared solutions were vortexed until a homogeneous solution was obtained, resulting in the preparation of the substrate solution. A fresh substrate solution was prepared and used for each set of experiments. When the activity of the free enzyme was determined, 0.1 mL of enzyme solution (1.5 mg/mL) was mixed with 1 mL of Tris-HCl buffer solution (50 mM, pH 8.0) and 1 mL of substrate solution. After the reaction mixture was incubated at 37 °C for 15 min, the enzymatic reaction was stopped by adding 0.1 mL of 0.1 M Na_2_CO_3_. In order to determine the enzymatic activity of the immobilized enzyme, PPL immobilized rPET/CA nanofiber was used instead of the free enzyme. Lipase catalyzes the hydrolysis of pNPP; it produces *p*-nitrophenol (pNP) and an inorganic phosphate. The pNP has a yellow color, and the lipase activity was measured spectrophotometrically at 410 nm. The definition of 1U lipase activity is the hydrolysis of 1 μmol pNPP per minute under specified analysis conditions at 37 °C.^93^ The Bradford method was used to determine the amount of protein for the free and immobilized enzymes.^94^ Furthermore, the amount of loaded protein was determined using the same method by calculating the difference between the initial amount of protein and the protein content in the supernatants after immobilization.

### 3.6 Biochemical Characterization of Free and Immobilized PPL

#### 3.6.1 Optimization of pH and Temperature

In order to determine the optimum pH value for both free PPL and PPL immobilized rPET/CA nanofibers, activity measurements were conducted using substrate solutions prepared with sodium phosphate, citrate, Tris-HCI, and sodium acetate buffer (50 mM) solutions at various pH values ranging from 3 to 10. Activity measurements were performed for both free PPL and PPL immobilized. The substrate solution prepared using 50 mM Tris-HCl (pH 8.0) was used, and activity measurements of free PPL and PPL immobilized rPET/CA nanofibers were made at temperatures between 20 and 60 °C, and optimum temperature values were determined for both forms.

#### 3.6.2 Assessment of pH and Thermal Stability

In order to determine the pH stability properties of the free PPL, activity measurements were made using PPL solutions prepared with sodium phosphate, citrate, Tris-HCI, and sodium acetate buffers (50 mM) with pH varying between 3 and 10. To investigate the pH stability properties of the immobilized PPL, the PPL immobilized rPET/CA nanofiber was exposed to buffer solutions with pH values ranging from 3.0 to 10.0 for a duration of 1 h. Following the incubation, the activity of the immobilized PPL was measured to assess its stability under different pH conditions. To assess the thermal stability of both free PPL and PPL immobilized rPET/CA nanofibers, the enzymes were subjected to elevated temperatures of 50, 60, and 70 °C for a period of 120 min (the substrate solution prepared using 50 mM Tris-HCl (pH 8.0) was used). At regular intervals of 10 min, the PPL activities were measured to monitor any changes in enzyme activity over time.

#### 3.6.3 Investigation of Kinetic Parameters

To determine the Michaelis–Menten constant (*K*_m_) and maximum velocity (*V*_max_) of free PPL and PPL immobilized rPET/CA nanofiber, PPL activity was measured using substrate solutions (pNPP) prepared at different concentrations ranging from 0.08 to 0.8 mM. The obtained data were analyzed by the Lineweaver–Burk plot to calculate *K*_m_ and *V*_max_ values.

#### 3.6.4 Storage Capacity

In the storage capacity experiments, the activity of both free PPL and PPL immobilized rPET/CA nanofibers was measured at regular time intervals over a period of 30 days. The measurements were conducted at 25 °C (room temperature).

#### 3.6.5 Reusability

To determine the number of reuses of the PPL immobilized rPET/CA nanofiber, the PPL activity was measured for a total of 14 cycles. In each cycle, the immobilized enzyme was used to catalyze the reaction and the reaction rate was determined. After each cycle, the immobilized enzyme was washed with 50 mM Tris-HCl (pH 8.0) buffer solution, and the activity was measured again in a subsequent cycle.

## 4 Conclusions

This study presents a pioneering approach to address both plastic recycling and enzyme immobilization challenges. By utilizing recycled poly(ethylene terephthalate) (rPET) from wastewater bottles and CA, we successfully fabricated nanofiber carriers for immobilized PPL. It was observed that rPET/CA nanofibers produced by electrospinning were an extremely suitable carrier for enzyme immobilization due to their high surface area properties. Additionally, it was found that PPL immobilized rPET/CA nanofibers showed activity higher than that of free PPL, especially at high temperatures and acidic conditions. It can be said that rPET/CA nanofibers act as a protective sheath for PPL molecules after the immobilization process, and therefore, the immobilized enzyme shows higher performance under extreme conditions. Moreover, due to their properties such as improved stability, specificity, and reusability, PPL immobilized rPET/CA nanofibers offer a versatile and effective platform for biocatalysis and wastewater treatment.

As a result, this study showed that by combining waste PET bottles with other polymers, electrospinning nanofibers that have different properties and can be used in different applications can be produced. Since electrospinning offers a simpler, more cost-effective, faster, and more sustainable method than other nanofiber production methods, it can be said that it will support the reduction and sustainability of plastic waste because of the nanofiber produced using plastic waste. This research offers the opportunity to produce nanofibers from wastewater bottles by the electrospinning method as well as to develop a new carrier for PPL immobilization. The fabricated rPET/CA nanofibers offer a promising platform for various biocatalytic applications including wastewater treatment, biodegradable material production, and pharmaceutical processes. This study underscores the importance of exploring unconventional materials and approaches to address contemporary environmental and biotechnological challenges.
